# Anti-Inflammatory Effect of *Turbo cornutus* Viscera Ethanolic Extract against Lipopolysaccharide-Stimulated Inflammatory Response via the Regulation of the JNK/NF-kB Signaling Pathway in Murine Macrophage RAW 264.7 Cells and a Zebrafish Model: A Preliminary Study

**DOI:** 10.3390/foods11030364

**Published:** 2022-01-27

**Authors:** Eun-A Kim, Nalae Kang, Junseong Kim, Hye-Won Yang, Ginnae Ahn, Soo-Jin Heo

**Affiliations:** 1 Jeju Marine Research Center, Korea Institute of Ocean Science & Technology (KIOST), Jeju 63349, Korea; euna0718@kiost.ac.kr (E.-A.K.); nalae1207@kiost.ac.kr (N.K.); junseong@kiost.ac.kr (J.K.); 2 Department of Marine Life Sciences, Jeju National University, Jeju 63243, Korea; koty221@naver.com; 3 Department of Food Technology and Nutrition, Chonnam National University, Yeosu 59626, Korea; 4 Department of Marine Biology, University of Science and Technology, Daejeon 34113, Korea

**Keywords:** *Turbo cornutus*, viscera, anti-inflammatory, RAW264.7 cells, zebrafish

## Abstract

*Turbo cornutus*, the horned turban sea snail, is found along the intertidal and basaltic shorelines and is an important fishery resource of Jeju Island. In this study, we performed a preliminary study on anti-inflammatory effect of 70% ethanol extract obtained from *T. cornutus* viscera (TVE) on lipopolysaccharide (LPS)-stimulated RAW264.7 cells in vitro and zebrafish embryos in vivo. TVE reduced the production of LPS-stimulated nitric oxide (NO) and prostaglandin E2 (PGE2) without any toxic effects. TVE also decreased the protein expression of LPS-induced inducible NO synthase and cyclooxygenase-2 and suppressed the production of pro-inflammatory cytokines, including tumor necrosis factor-α, interleukin (IL)-6, and IL-1β. Furthermore, mechanistic studies indicated that TVE suppressed c-Jun N-terminal kinase phosphorylation and nuclear factor-kB activation. In zebrafish embryos, TVE did not show developmental toxicity based on the survival rate and cell death findings. In LPS-stimulated zebrafish embryos, TVE suppressed NO production and cell death. In conclusion, the result from this preliminary study showed TVE has a potential anti-inflammatory property that can be exploited as a functional food ingredient.

## 1. Introduction

The spiny top shell *Turbo cornutus* (Phylum Mullusca, Class Gastropoda, Order Trochida) is distributed throughout the coastal regions of South Korea, Japan, China, and Taiwan, and it inhabits the rocky intertidal zone within a water depth of 20 m [1]. *T. cornutus* is an edible gastropod species, and it commands a high economic value in Asia [2]. In South Korea, *T. cornutus* is used as an important fishery resource and is a major gastropod in the marine industry [3]. The highest amount of *T. cornutus* (83% of the total national fishery production) is produced in Jeju Island, South Korea, and it is a major source of income for Haenyeo (female divers whose practices are listed as a cultural heritage practice by the United Nations Educational, Scientific, and Cultural Organization). *T. cornutus* is an extremely important marine resource, and studies have evaluated the marine environment of its habitat, resource biology, and population and immune-associated activities of hemocytes in reproductive biology and physiology [1,2,4]. However, the plausible biological mechanism related to the functional activities of *T. cornutus* has not been elucidated yet.

The fishing industry generates several by-products, including viscera, skin, eggs, and gonads, and these products are discarded as industrial waste [5,6]. However, these by-products may have various applications as functional food ingredients [5]. Among the by-products, viscera contain a high amount of proteins and lipids and exhibit several valuable effects, including anti-oxidant, cytoprotective, and anti-inflammatory activities of abalone, anti-oxidant activity of sea snails as well as anti-oxidant, and immune-enhancing activities of sea cucumber [5,6,7,8,9,10].

Inflammation is a natural protective response of the body tissues to various external stimuli, such as invading pathogens, tissue damage, and irritants [11,12]. However, uncontrolled inflammation inflicts damage to the body and can lead to obesity, diabetes arthritis, asthma, or other diseases [13,14]. Macrophages play an important role in host defenses against noxious substances and subsequently initiate and regulate inflammatory responses [15,16]. The murine macrophage cell line RAW 264.7 has been widely used to study the anti-inflammatory effects. During an inflammatory response, murine macrophages promote the endocytosis of bacterial debris (such as the major gram-negative bacterial component lipopolysaccharide [LPS]) and subsequently produce nitric oxide (NO), prostaglandin E2 (PGE2), and pro-inflammatory cytokines, thereby expanding the local inflammatory response [14,15]. The activation of both nuclear factor-kB (NF-kB) and mitogen-activated protein kinase (MAPK) family members, including extracellular signal-regulated kinase (ERK), c-Jun N-terminal kinase (JNK), and p38 are important for the production of pro-inflammatory factors and are triggered during inflammation under LPS induction in macrophages [13,17]. These mechanisms have been widely explored for investigating the major target of anti-inflammatory activity.

Zebrafish (*Danio rerio*) has been used as a major laboratory animal model in diverse fields, including genetics, toxicity, biology, and drug discovery research, owing to their extrauterine development, optical clarity, high fecundity, phenotypic similarity to human tissues, and ease of genetic manipulation [18,19,20]. Zebrafish larvae have an innate immune system that comprises neutrophils, macrophages, eosinophils, and mast cells [21]. Hence, zebrafish have been utilized in the discovery of an anti-inflammatory substance that can be used in drugs and/or health functional foods [14,21,22,23]. In the present study, we used zebrafish embryos to confirm the anti-inflammatory activity of TVE.

The present study aimed to explore the anti-inflammatory effects of 70% ethanol extract obtained from *T. cornutus* (TVE) on LPS-stimulated murine macrophage RAW264.7 cells in vitro and zebrafish embryos in vivo.

## 2. Materials and Methods

### 2.1. Preparation of TVE

#### 2.1.1. Extraction of TVE

*T. cornutus* were purchased from the fishing village market of Jeju Island, South Korea. Its surface was washed with running water to remove salt, sand, and foreign materials. After removing the shells, each specimen was divided into the muscle and viscera. The viscera were then thoroughly washed using tap water to remove foreign materials and stored at −20 °C. The viscera were freeze-dried and ground to a powder. The powdered viscera (5 g) was dissolved using one liter of 70% ethanol in a sonicator for 1 h at room temperature; this was repeated three times, and the solution was then filtered by vacuum filters. Finally, TVE was concentrated using a rotary evaporator and lyophilized.

#### 2.1.2. Microbiological Analysis of TVE

The microbial analysis of TVE was performed using standard plate count and dry rehydratable film methods for detection of total bacterial counts and Escherichia coli according to the standard method of the Korean Food Code [24]. Analysis of the total bacterial counts indicated that 10 mL TVE was added to a 90 mL sterile saline solution and homogenized in a homogenizer for 2 min at room temperature. 1 mL of sample was mixed with 15 mL of the plate count agar (Merck, Darmstadt, Germany) and then incubated at 37 °C for 48 h. The amount of bacteria is expressed as colony forming units (CFU/mL). For the quantification of Escherichia coli, TVE plated onto dry rehydratable film (3M Petrifilm TM, 3M Microbiology products, St. Paul, MN, USA), and then incubated for 24 h at 35 °C and count blue colonies with entrapped gas. As well as detection of bacteria and fungal was assessed using the PCR Bacteria Test Kit (PromoKine, Heidelberg, Germany), and Fungal rDNA PCR Kit (Takara Bio, Tokyo, Japan) according to the manufacturer’s instructions. PCR (Takara, Kusatsu, Japan) was performed with positive and negative controls provided in the kits using 1.5% agarose gel electrophoresis.

### 2.2. In Vitro Cell Experiments

#### 2.2.1. Measurement of NO Production and Cell Viability Assay

RAW 264.7 cells are monocyte/macrophage-like cells, originating from Abelson murine leukemia virus-induced tumors in BALB/c mice (KCLB; Seoul, Korea). Murine macrophage RAW264.7 cells were cultured in Dulbecco’s Modified Eagles Media supplemented with 10% FBS, 100 µg/mL streptomycin, and 100 unit/mL penicillin at 37 °C in an incubator with a humidified atmosphere of 5% CO_2_. RAW264.7 cells (1.5 × 10^5^ cells/mL) were seeded in 24-well plates. After 16 h, the cells in various concentrations were treated with 50, 100, or 200 μg/mL of TVE and then incubated for 1 h. Then, LPS (1 µg/mL) was added for cotreatment, and the cells were incubated for another 24 h. Cell viability was evaluated via the MTT (3-[4,5-Dimethylthiazol-2-yl]-2,5-diphenyltetrazolium bromide) tetrazolium salt colorimetric assay. MTT solution was added to each well for 3 h. After, culture supernatants were removed and DMSO (dimethyl sulfoxide) added to dissolve the formazan crystals. NO production was evaluated via the Griess assay. Griess reagent and culture supernatants were mixed for 10 min in dark. The cell viability and NO production were measured at 570 nm and 540 nm using a microplate reader, respectively. Experiments were performed as described previous study [14].

#### 2.2.2. Measurement of PGE2 and Pro-Inflammatory Cytokine Production

The first part of this experiment was performed as described in Section 2.2.1. The culture supernatants were collected after 24 h of culture. PGE2 and pro-inflammatory cytokine (tumor necrosis factor-α [TNF-α], interleukin [IL]-6, IL-1β) were determined using mouse ELISA kits (R&D Systems, Inc., St. Louis, MO, USA) for PGE2 production, and a mouse ELISA kit (Biosciences, San Diego, CA, USA) for pro-inflammatory cytokine production as per the manufacturer’s instructions. The optical density of the solution was determined by measuring the absorbance at 450 nm using a microplate reader.

#### 2.2.3. Western Blots

RAW264.7 cells were seeded in 6-well plates. The cells were treated with 50, 100, or 200 μg/mL of TVE for 1 h and sensitized with LPS (1 µg/mL) for another 24 h or 15 min of incubation. The cells were subsequently harvested and washed twice with ice PBS (phosphate buffered saline) and pelleted by centrifugation for 20 min at 12,000 rpm. The protein concentration was measured using a BCA^TM^ protein assay kit (Thermo Scientific, Waltham, MA, USA) according to manufacturer’s instructions. β-actin, inducible NO synthase [iNOS], cyclooxygenase-2 [COX-2], Ik-B, phospho (p)-p65, ERK, p-ERK, JNK, p-JNK, p-38, and p-p38 were used as the primary antibodies (1:1000 dilution, Cell Signaling Technology, Beverly, MA, USA). The HRP-conjugated anti-mouse IgG and anti-rabbit IgG were used secondary antibodies (1:5000 dilution, Santa Cruz Biotechnology, CA, USA). A densitometric analysis was performed using ImageJ to quantify protein expression [25].

### 2.3. In Vitro Zebrafish Experiments

#### 2.3.1. Assessment of the Survival Rate and Morphology

Zebrafish were cultured and maintained as per conditions described previously [18]. Zebrafish (one female and two males) were mated and spawning was stimulated by the onset of light. Naturally spawning embryos were transferred to petri dishes containing embryo media within 30 min. The embryos of 7–9 h post-fertilization were transferred to 12-well plates, and TVE was added to each well (15 embryos/well). After 1 h, LPS was added to the wells until 3 days post-fertilization (dpf). The survival rate was evaluated at 7 dpf, and morphology was observed under a microscope equipped with DS-Fi1c digital camera (Nikon, Tokyo, Japan), which was used to capture morphological images, at 2 and 3 dpf. The zebrafish experiments were performed in accordance with the regulation of the Animal Care and Use Committee of the Animal Center of Jeju National University (2020-0049).

#### 2.3.2. Measurement of Cell Death and NO Production

At 3 dpf, zebrafish was transferred to each well of a 24-well plate, treated with 5 μM DAF-FM-DA solution (Sigma-Aldrich, St. Louis, MO, USA), and incubated for 2 h in the dark for measuring NO production. For detecting cell death, the fish were treated with 7 μg/mL acridine orange solution (Sigma-Aldrich, St. Louis, MO, USA) and incubated for 30 min in the dark. After incubation, the zebrafish were rinsed to remove the fluorescence solution using fresh embryo media and anesthetized with 0.003% MS-222 (tricaine methane-sulfonate, Sigma-Aldrich, St. Louis, MO, USA) before observation. The embryos were also photographed under a fluorescence microscope equipped with a DS-Fi1c digital camera (Nikon, Tokyo, Japan) at 3 dpf. The fluorescence intensity of individual larva was quantified using Image J.

### 2.4. Statistical Analysis

All experiments were performed in triplicate, and the data were denoted as means ± standard deviation. Significant differences between the means of the parameters were evaluated using a one-way analysis of variance, followed by Tukey’s multiple range test. All analyses were performed using IBM SPSS (* *p* < 0.5, ** *p* < 0.01).

## 3. Results

### 3.1. TVE Inhibited the NO and PGE2 Production Stimulated by LPS in RAW264.7 Cells

**Figure 1:**
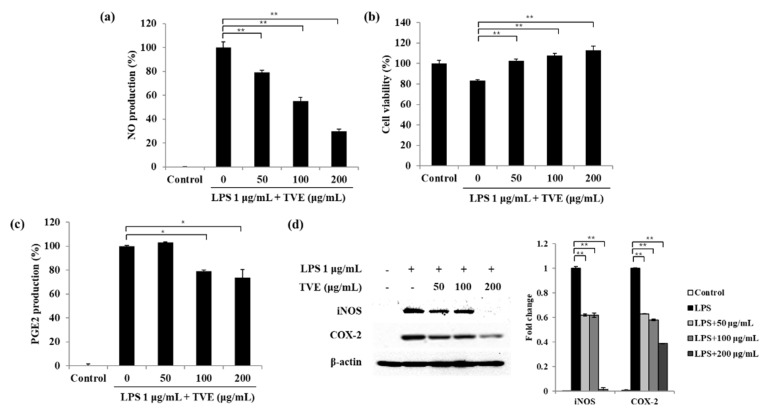
Effect of *Turbo cornutus* viscera ethanol extract (TVE) on LPS -stimulated inflammatory mediators in RAW 264.7 cells via (**a**) NO production, (**b**) cell viability, (**c**) PGE2 production, and (**d**) iNOS and COX−2 protein levels. The data are expressed as the mean ± standard deviation of triplicate experiments. * *p* < 0.05, ** *p* < 0.01 indicate values compared with the only LPS-stimulated group.

As the key inflammatory targets, NO and PGE2 are considered inflammatory indicators in animal models [26]. At the various concentrations of TVE ranging from 50 to 200 µg/mL, NO production was markedly decreased by 20.92%, 44.86%, and 70.21% (for 50, 100, and 200 µg/mL, respectively) compared with that in the only LPS-stimulated group without any cytotoxicity (Figure 1a,b). As shown in Figure 1c, the only LPS-stimulated group had considerably increased PGE2 production. However, TVE inhibited 20.77% and 26.39% of PGE2 production at 100 and 200 µg/mL compared with the only LPS-stimulated group. We then measured the protein levels of iNOS and COX-2 (Figure 1d). The protein levels of iNOS and COX-2 were upregulated after LPS treatment, but TVE significantly downregulated their levels. These results suggest that TVE inhibits NO and PGE2 production by downregulating both iNOS and COX-2 protein levels in LPS-stimulated RAW264.7 cells.

### 3.2. TVE Suppressed the Pro-Inflammatory Cytokines Stimulated by LPS in RAW264.7 Cells

**Figure 2:**
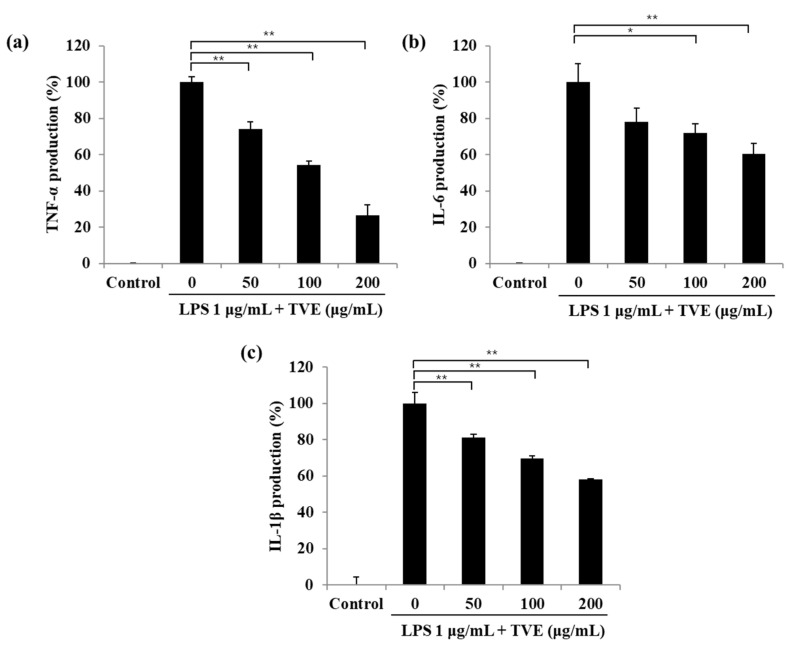
Inhibitory effect of *Turbo cornutus* viscera ethanol extract (TVE) on LPS-induced (**a**) TNF-α, (**b**) IL-6, and (**c**) IL-1β production in RAW 264.7 cells via ELISA. The data are expressed as the mean ± standard deviation of triplicate experiments. * *p* < 0.05, ** *p* < 0.01 indicate values compared with the only LPS-stimulated group.

As shown in Figure 1, TVE suppressed NO and PGE2 production as well as iNOS and COX-2 protein levels. Therefore, we evaluated whether TVE affects pro-inflammatory cytokine production against LPS treatment (Figure 2). Pro-inflammatory cytokines such as TNF-α, IL-6, and IL-1β are mediated via the association of iNOS and COX-2 [26]. LPS treatment significantly induced TNF-α, IL-6, and IL-1β production compared with the control group. However, TVE significantly decreased TNF-α production by 74.31%, 54.24%, and 26.76% (Figure 2a); IL-6 production by 78.12%, 72.06%, and 60.55% (Figure 2b); and IL-1β production 81.27%, 69.62%, and 58.12% (Figure 2c) at 50, 100, and 200 mg/mL concentrations, respectively.

### 3.3. TVE Downregulated the MAPK and NF-kB Signaling Pathways in LPS-Induced RAW264.7 Cells

**Figure 3:**
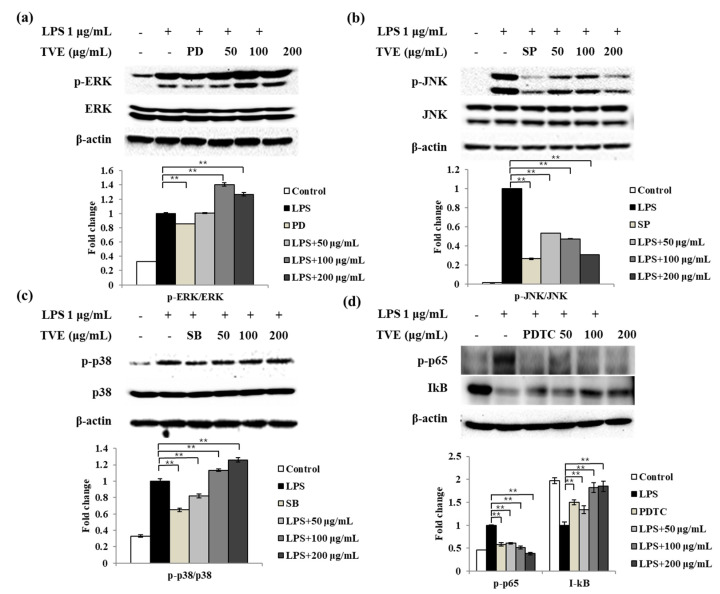
Inhibitory effect of TVE on the LPS-stimulated MAPK and NF-kB signaling pathways in RAW 264.7 cells. Protein levels of (**a**) p−ERK and ERK, (**b**) p−JNK and JNK, (**c**) p−p38 and p38, and (**d**) I-kB and p−p65 were analyzed using western blot. Pharmacological inhibitors; PD (PD98059, ERK inhibitor), SP (SP600125, JNK inhibitor), SB (SB203580, p38 inhibitor), and PDTC (Pyrrolidine dithiocarbamate, NF−kB inhibitor).”The data are expressed as the mean ± standard deviation of triplicate experiments. ** *p* < 0.01 indicate values compared with the only LPS-stimulated group.

Next, we studied the anti-inflammatory signaling mechanisms of MAPK and NF-kB, which play pivotal roles during inflammatory responses [27]. LPS treatment increased the phosphorylation of ERK, JNK, and p38 in the MAPK signaling pathway (Figure 3a–c). As indicated in Figure 3b, SP (SP600125) is widely used to inhibit JNK-mediated activation, which was downregulated in LPS-induced RAW264.7 cells. The level of phosphorylated JNK was significantly reduced after TVE treatment. However, TVE did not downregulate the level of phosphorylated ERK and p38 in LPS-induced RAW264.7 cells. Therefore, TVE inhibits LPS-induced inflammatory responses by suppressing the MAPK-associated JNK pathway. The levels of the phosphorylated p65 subunit of NF-kB and IkB in the NF-kB signaling mechanism are presented in Figure 3d. LPS stimulation downregulated IkB and upregulated p65 phosphorylation in the cytosol compared with that in the control group. However, treatment with TVE increased IkB protein levels and decreased p-p65 protein levels. Therefore, TVE regulated the level of IkB and p-p65 protein levels. These results suggest that TVE improves the inflammatory response by suppressing the JNK and NF-kB signaling mechanism in LPS-stimulated RAW264.7 cells.

### 3.4. TVE Has No Toxic Effects in LPS-Stimulated Zebrafish

**Figure 4:**
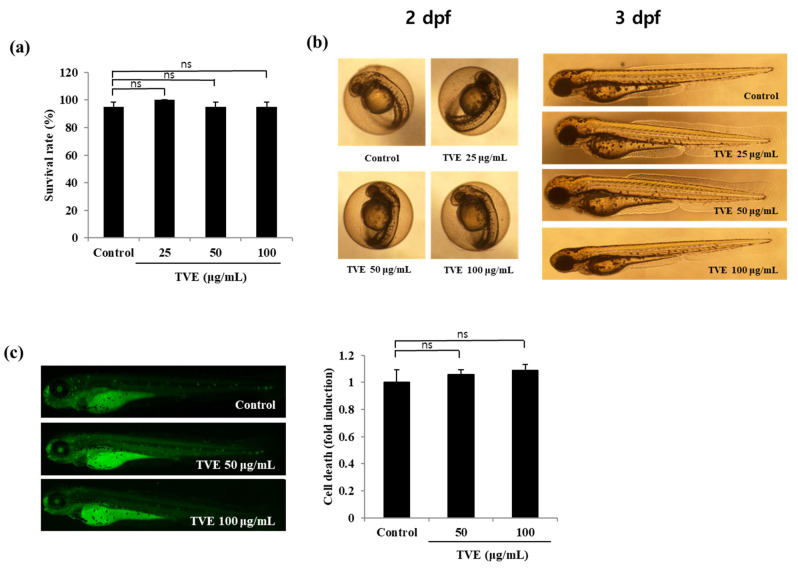
Toxicity of TVE in zebrafish via (**a**) survival rate, (**b**) morphology, and (**c**) cell death. The data are expressed as the mean ± standard deviation of triplicate experiments. *p* values were not significant (ns).

The zebrafish is a useful animal experimental model in toxicological studies [28]. To confirm the toxicity of TVE, we monitored the survival rates, morphology, and cell death of zebrafish. The survival rate of zebrafish after TVE treatments was >95%, with no significant difference between the control and treatment groups at 7 dpf (Figure 4a). The representative images of zebrafish at 2 and 3 dpf are shown in Figure 4b. The TVE-exposed groups did not differ from the control groups with respect to the body length and yolk sac size. Cell death as detected via fluorescence staining is shown in Figure 4c. The developmental toxicity of TVE was not detected at concentrations ranging from 25 to 100 μg/mL.

### 3.5. TVE Reduced NO Production in LPS-Induced Zebrafish

**Figure 5:**
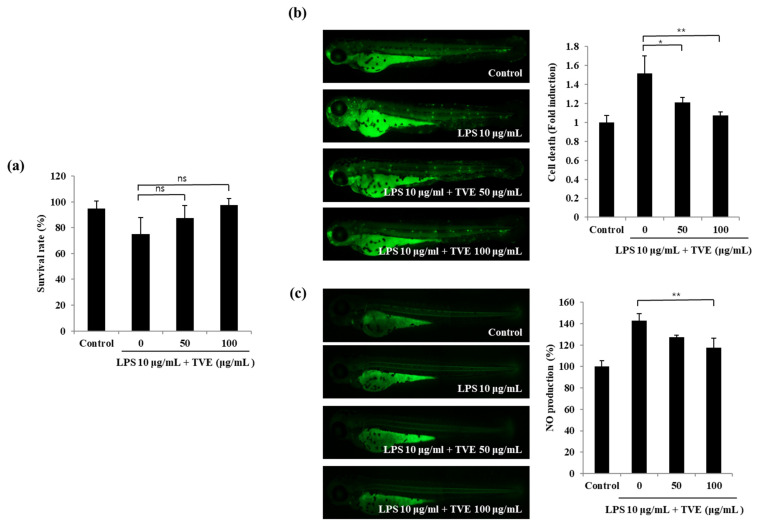
Inhibitory effect of TVE on LPS-stimulated inflammatory mediators in the zebrafish model via (**a**) survival rate, (**b**) cell death, and (**c**) NO production. The data are expressed as the mean ± standard deviation of triplicate experiments. * *p* < 0.05, ** *p* < 0.01 indicate values compared with the only LPS-stimulated group and ns indicates not significant.

The anti-inflammatory effect of TVE in zebrafish was evaluated via its survival rate, cell death, and NO production. The LPS-treated group showed reduced survival; however, TVE increased the survival rate, which was similar to that of the control group (Figure 5a). Moreover, the fluorescence images revealed that the zebrafish exposed to TVE with LPS had a marked reduction in cell death (Figure 5b). Next, NO production was increased after LPS stimulation and decreased with the increase in TVE concentrations (Figure 5c). These results suggest that TVE treatment suppresses the inflammatory mediators in LPS-stimulated zebrafish.

## 4. Discussion

*T. cornutus* represents one of the most common edible gastropod species. Normally, only the muscle part is consumed, whereas most of the viscera part is discarded as fishery manufacturing waste, causing environmental pollution [4,5]. Therefore, extensive research is required to find applications for the viscera. A recently published study revealed the antioxidant properties of *T. cornutus* viscera in H_2_O_2_-induced HepG2 cells and purified peptides via enzymatic hydrolysis [5]. The health-promoting effect of *T. cornutus* viscera associated with various functional activities has rarely been reported. Moreover, TVE was microbiologically safe according to microbiological analysis. TVE did not detect Escherichia coli and fungal (Supplementary Table S1, Figure S1). The levels of total bacteria on TVE was 7 CFU/mL, it was detected below regulation limit of the Korean government guidelines (Regulations Concerning Recognition of Functional Ingredients and Standards and Specifications for Health Functional Foods), and TVE did not detect bacteria via PCR assay (Supplementary Table S1, Figure S1). Therefore, we prepared TVE and confirmed its functionality for increased usability. However, in order to use TVE as an ingredient for functional food that can be consumed by humans in the manufacturing industry, it will be necessary to require microbiological hazards identification and sterilization process because TVE is a substance derived from the viscera.

Inflammation is an important nonspecific protective response of body tissues to harmful stimulation and is associated with many diseases [13,14,29]. Therefore, in our study, we focused on anti-inflammatory activity. This study was conducted to identify applications in which *T. cornutus* viscera can be utilized as a valuable material and/or additive in the functional food industry.

NO and PEG2 are key molecules that regulate inflammatory activity via iNOS and COX-2 in animal models, respectively [30,31]. Thus, suppressing the production of these inflammatory mediators serves a fundamental purpose in the treatment of inflammatory diseases. The present study results showed that TVE markedly decreased LPS-stimulated NO production in both RAW264.7 cells and zebrafish without toxicity. TVE also inhibited the production of PGE2 and significantly reduced the expression levels of iNOS and COX-2 proteins in LPS-stimulated RAW 264.7 cells. These results suggest that TVE effectively inhibits the production of inflammatory mediators. Next, we evaluated whether LPS-induced macrophages that induce the secretion of the pro-inflammatory cytokines IL-6, IL-1β, and TNF-α are suppressed by TVE. TNF-α functions as a critical cytokine in pathological damage associated with inflammation and can induce the release of IL-6 and IL-1β, which are the key pathogenic elements in various inflammatory disorders [14,15,30]. The stimulation of these cytokines is believed to be a capable therapeutic approach for the clinical treatment of inflammatory disorders [11]. The study results showed that TVE effectively decreases the pro-inflammatory cytokine expression in activated RAW264.7 cells. Therefore, TVE can be used as an additive in the prevention and treatment of inflammatory-related disease via the inhibition of pro-inflammatory cytokines.

For the discovery of new anti-inflammatory drugs, the study of signaling pathways plays a crucial role. A key target for exploring the mechanism of LPS-stimulated inflammatory response is to study anti-inflammatory mediators that inhibit the MAPK and NF-kB signaling pathways [13,14]. The MAPK signaling pathway is involved in many biological processes, including inflammation, apoptosis, and proliferation, and is known to activate iNOS and COX-2 expression along with pro-inflammatory cytokine production in LPS-induced macrophages [14,30]. The NF-kB signaling pathway plays a significant role in cell survival, DNA transcription, and cytokine production, and NF-kB is activated by the degradation of IkB proteins, which lead to the continuous nuclear translocation of NF-KB subunits [11,12]. Subsequently, the regulation of the immune response to infection adjusts the expression of inflammatory factors, including iNOS, COX-2, IL-6, IL-1β, and TNF-α [12,30]. To demonstrate the inhibitory activity of TVE on the pro-inflammatory cytokines and mediators, MAPK and NF-kB levels in LPS-activated macrophages were evaluated. LPS inherently activated MAPK phosphorylation, including that of ERK, JNK, and p38. However, TVE treatment selectively inhibited the phosphorylation of JNK activation without affecting ERK and p38 activation in a concentration-dependent manner. The JNK signaling pathway has been implicated in the development of inflammatory responses in various conditions, which leads to organ dysfunction [32]. It has also been reported that the suppression of JNK activation reduces LPS-stimulated inflammatory responses in RAW264.7 cells [33,34]. TVE attenuated the LPS-induced degradation of IkB and phosphorylation of p65. Thus, the present data demonstrate that TVE produces anti-inflammatory effects in LPS-stimulated RAW264.7 cells by inhibiting the JNK/NF-kB signaling pathway. Moreover, these data suggest that TVE is an important integrator of inflammation-inducible signaling. Therefore, we suggest that TVE could be useful as a supplement with bioactive properties for anti-inflammation. However, many of the factors used for murine macrophages have not translated to human macrophages [35]. Therefore, depth of animal trials and clinical trials are needed to prove that TVE is a substance that can be consumed by humans.

Abalone, a marine gastropod, is a valuable fishery and food product. However, it is also a source of visceral waste, which is an environmental contamination source [9,10,36]. Abalone intestine has anti-inflammatory activities against NO production via iNOS expression in LPS-treated macrophages, and the abalone viscera extract derived via digestion with papain and bromelain decreased LPS-stimulated NO and pro-inflammatory cytokine production via the MAPK signaling pathway in RAW 264.7 cells [9,10]. Furthermore, purified peptides derived from the abalone gastrointestinal tract attenuated the production of NO, iNOS, and pro-inflammatory cytokine via the downregulation of the MAPK signaling pathway in LPS-induced RAW264.7 cells [37]. The viscera of marine organisms have a high lipid content, including polyunsaturated fatty acids (PUFA) (such as eicosapentaenoic acid [EPA] and docosahexaenoic acid [DHA]), but most are waste products derived from common seafood with significant anti-inflammatory activities [37]. Saito and Aono reported that the lipid content in *T. cornitus* viscera ranged from 1.7% to 4.0% of wet weight and that the major PUFA is arachidonic acid (ARA), EPA, and docosapentaenoic acid (n-3 DPA) along with a low content of DHA [38]. n-3 DPA is not readily available, and ARA is considered the most important for infants [38]. In particular, DPA is a substrate for the synthesis of resolvins, maresins, and protectins. Resolvins are specialized pro-resolving mediators derived from EPA, DHA, and DPA by the action of lipoxygenases; maresins are DHA and DPA derived metabolites produced by macrophages; and protectins are derived from DHA and DPA. All these specialized pro-resolving mediators have potent anti-inflammatory activities [39]. Therefore, they may have important roles in human health, including in the promotion of anti-inflammatory mechanisms and protection of different organs [39,40]. *T. cornitus* viscera, therefore, is an important source of valuable seafood because of its possible health benefits.

The viscera of *T. cornutus* have rich active materials; however, further research is needed to isolate and investigate the properties and mechanisms of its active components.

## 5. Conclusions

The present study results demonstrated that TVE treatment suppresses the LPS-stimulated production of NO, PGE2, and cytokines by inhibiting JNK phosphorylation and blocking NF-kB activation in RAW264.7 cells. TVE treatment increased the survival rate as well as decreased NO production and cell death compared with the only LPS-stimulated zebrafish model. These results are preliminary studies confirming the anti-inflammatory activity of *T. cornutus* viscera. Therefore, in order to utilize TVE as an ingredient for functional foods that can be consumed by humans, it is required further experiments such as various safety evaluations, clinical trials, isolation of active components, etc. Afterward, the anti-inflammatory effects of *T. cornutus* viscera by-products derived from the fishing industry may utilize the possibility of their highly valuable utilization and application as functional foods.

## Supplementary Materials

The following supporting information can be downloaded at: https://www.mdpi.com/article/10.3390/foods11030364/s1, Figure S1: Detection of (a) bacteria and (b) fungal in *Turbo cornutus* viscera ethanol extract (TVE), Table S1: Detection of bacteria counts and Escherichia coli in Turbo cornutus viscera ethanol extract (TVE).

## References

[1] Kwon D.H., Chang D.S., Lee S.J., Koo J.H., Kim B.Y. (2010). Stock Assessment and Management of Turban shell, Turbo (Batillus) cornutus Lightfoot, 1786 in Jeju Coastal waters, Korea. Korean J. Malacol..

[2] Donaghy L., Hong H.K., Lambert C., Park H.S., Shim W.J., Choi K.-S. (2010). First characterisation of the populations and immune-related activities of hemocytes from two edible gastropod species, the disk abalone, *Haliotis discus discus* and the spiny top shell, *Turbo cornutus*. Fish. Shellfish Immunol..

[3] Jung G.K., Park J.J., Ju S.M., Jin Y.G., Lee J.S. (2007). Ovarian structure and oogenesis of the spiny top shell, *Batillus cornutus* (Lightfoot, 1786) (Gastropoda: Turbinidae). Korean J. Malacol..

[4] Matoto S.V., Shimizu T., Mita H., Tsuchiya K., Segawa S. (2002). Reproduction and metabolism of Turbo (Batillus) cornutus in Chiba, Japan. Fish. Res..

[5] Kang N., Kim E.A., Kim J., Lee S.H., Heo S.J. (2021). Identifying potential antioxidant properties from the viscera of sea snails (*Turbo cornutus*). Mar. Drugs.

[6] Je J.Y., Park S.Y., Hwang J.Y., Ahn C.B. (2015). Amino acid composition and in vitro antioxidant and cytoprotective activity of abalone viscera hydrolysate. J. Funct. Foods.

[7] Yang D., Lin F., Hung Y., Ye J., Xiao M. (2020). Separation, purification, structural analysis and immune-enhancing activity of sulfated polysaccharide isolated form sea cucumber viscera. Int. J. Biol. Macromol..

[8] Zhou D.Y., Zhu B.W., Qiao L., Wu H.T., Li D.M., Yang J.F., Murata Y. (2012). In vitro antioxidant activity of enzymatic hydrolysates prepared form abalone (*Haliotis discus* hannai Ino) viscera. Food Bioprod. Process..

[9] Qian Z.J., Kim S.A., Lee J.S., Kim H.J., Choi I.W., Jung W.K. (2012). The antioxidant and anti-inflammatory effects of abalone intestine digest, *Haliotis discus* hannai in RAW 264.7 macrophages. Biotechnol. Bioprocess. Eng..

[10] Qian Z.J., Ryu B., Park W.S., Choi I., Jung W.K.J. (2016). Inhibitory effects and molecular mechanism of an anti-inflammatory peptide isolated from intestine of abalone, *Haliotis discus* Hannai on LPS-induced cytokine production via the p-p38/p-JNK pathways in RAW264. 7 macrophages. J. Food Nutr. Res..

[11] Feng M., Wang X., Xiong H., Qiu T., Zhang H., Guo F., Jiang L., Sun Y. (2021). Anti-inflammatory effects of three selenium-enriched brown rice protein hydrolysates in LPS-induced RAW264. 7 macrophages via NF-κB/MAPKs signaling pathways. J. Funct. Foods.

[12] Wang L., Oh J.Y., Yang H.W., Fu X., Kim J.I., Jeon Y.J. (2021). Fucoidan isolated from the popular edible brown seaweed *Sargassum fusiforme* suppresses lipopolysaccharide-induced inflammation by blocking NF-κB signal pathway. J. Appl. Phycol..

[13] Cao J., Li Q., Shen X., Yao Y., Li L., Ma H. (2021). Dehydroepiandrosterone attenuates LPS-induced inflammatory responses via activation of Nrf2 in RAW264. 7 macrophages. Mol. Immunol..

[14] Kim E.A., Kim S.Y., Kim J., Oh J.Y., Kim H.S., Yoon W.J., Kang D.H., Heo S.J. (2019). Tuberatolide B isolated *from Sargassum macrocarpum* inhibited LPS-stimulated inflammatory response via MAPKs and NF-κB signaling pathway in RAW264. 7 cells and zebrafish model. J. Funct. Foods.

[15] Yang G., Lee K., Lee M., Ham I., Choi H.Y. (2012). Inhibition of lipopolysaccharide-induced nitric oxide and prostaglandin E2 production by chloroform fraction of *Cudrania tricuspidata* in RAW 264.7 macrophages. BMC Complementary Altern. Med..

[16] Kim K.N., Ko Y.J., Yang H.M., Ham Y.M., Roh S.W., Jeon Y.J., Ahn G., Kang M.C., Yoon W.J., Kim D. (2013). Anti-inflammatory effect of essential oil and its constituents from fingered citron (*Citrus nedica* L. var. sarcodactylis) through blocking JNK, ERK and NF-kB signaling pathways in LPS-activated RAW264.7 cells. Food Chem. Toxicol..

[17] Liu X., Yin S., Chen Y., Wu Y., Zheng W., Dong H., Bai Y., Qin Y., Li J., Feng S. (2018). LPS-induced proinflammatory cytokine expression in human airway epithelial cells and macrophages via NF-κB, STAT3 or AP-1 activation. Mol. Med. Rep..

[18] Kim E.A., Lee J.H., Heo S.J., Jeon Y.J. (2021). Saringosterol acetate isolated from *Hizikia fusiforme*, an edible brown alga, suppressed hepatocellular carcinoma growth and metastasis in a zebrafish xenograft model. Chem. Biol. Interact..

[19] Bradford Y.M., Toro S., Ramachandran S., Ruzicka L., Howe D.G., Eagle A., Kalita P., Martin R., Taylor Moxon S.A., Schaper K. (2017). Zebrafish models of human disease: Gaining insight into human disease at ZFIN. ILAR J..

[20] Mathias J.R., Dodd M.E., Walters K.B., Yoo S.K., Ranheim E.A., Huttenlocher A.J.D. (2009). Characterization of zebrafish larval inflammatory macrophages. Dev. Comp. Immunol..

[21] Lee S.H., Ko C.I., Jee Y., Jeong Y., Kim M., Kim J.S., Jeon Y.-J. (2013). Anti-inflammatory effect of fucoidan extracted from *Ecklonia cava* in zebrafish model. Carbohydr. Polym..

[22] Ko E.Y., Cho S.H., Kwon S.H., Eom C.Y., Jeong M.S., Lee W., Kim S.Y., Heo S.J., Ahn G., Lee K.P. (2017). The roles of NF-κB and ROS in regulation of pro-inflammatory mediators of inflammation induction in LPS-stimulated zebrafish embryos. Fish. Shellfish Immunol..

[23] De Sá Coutinho D., Pacheco M.T., Frozza R.L., Bernardi A. (2018). Anti-inflammatory effects of resveratrol: Mechanistic insights. Int. J. Mol. Sci..

[24] (2014). KFDA Food Code.

[25] Han E.J., Fernando I.P.S., Kim E.A., Kim J., Jung K., Kim S.Y., Cha S.H., Kim K.N., Heo S.J., Ahn G. (2020). 5-Bromo-3, 4-dihydroxybenzaldehyde from *Polysiphonia morrowii* attenuate IgE/BSA-stimulated mast cell activation and passive cutaneous anaphylaxis in mice. Biochem. Pharmacol..

[26] Jayawardena T.U., Sanjeewa K., Nagahawatta D., Lee H.G., Lu Y.A., Vaas A., Abeytunga D., Nanayakkara C., Lee D.S., Jeon Y.-J. (2020). Anti-Inflammatory Effects of Sulfated Polysaccharide from Sargassum Swartzii in Macrophages via Blocking TLR/NF-Κb Signal Transduction. Mar. Drugs.

[27] Li M.Y., Sun L., Niu X.T., Chen X.M., Tian J.X., Kong Y.D., Wang G.Q. (2019). Astaxanthin protects lipopolysaccharide-induced inflammatory response in *Channa argus* through inhibiting NF-κB and MAPKs signaling pathways. Fish. Shellfish Immunol..

[28] Chahardehi A.M., Arsad H., Lim V. (2020). Zebrafish as a successful animal model for screening toxicity of medicinal plants. Plants.

[29] Kim E.A., Kim S.Y., Ye B.R., Kim J., Ko S.C., Lee W.W., Kim K.N., Choi I.W., Jung W.K., Heo S.J. (2018). Anti-inflammatory effect of Apo-9′-fucoxanthinone via inhibition of MAPKs and NF-kB signaling pathway in LPS-stimulated RAW 264.7 macrophages and zebrafish model. Int. Immunopharmacol..

[30] Kim K.N., Ko S.C., Ye B.R., Kim M.S., Kim J., Ko E.Y., Cho S.H., Kim D., Heo S.J., Jung W.K. (2016). 5-Bromo-2-hydroxy-4-methyl-benzaldehyde inhibited LPS-induced production of pro-inflammatory mediators through the inactivation of ERK, p38, and NF-κB pathways in RAW 264.7 macrophages. Chem. Biol. Interact..

[31] Yoon W.J., Ham Y.M., Kim K.N., Park S.Y., Lee N.H., Hyun C.G., Lee W.J. (2009). Anti-inflammatory activity of brown alga *Dictyota dichotoma* in murine macrophage RAW 264.7 cells. J. Med. Plant Res..

[32] Vo V.A., Lee J.W., Park J.H., Kwon J.H., Lee H.J., Kim S.S., Kwon Y.S., Chun W. (2014). N-(p-Coumaryol)-Tryptamine suppresses the activation of JNK/c-Jun signaling pathway in LPS-challenged RAW264.5 cells. Biomol. Ther..

[33] Lee J.W., Kim N.H., Kim J.Y., Park J.H., Shin S.Y., Kwon Y.S., Lee H.J., Kim S.S., Chun W. (2013). Aromadendrin inhibits lipopolysaccjaride-induced nuclear translocation of NF-kB and phosphorylation of JNK in RAW264.7 macrophage cells. Biomol. Ther..

[34] Li L., Sapkota M., Kim S.W., Soh Y. (2015). Herbacetin inhibits inducible nitric oxide synthase via JNK and nuclear factor-kB in LPS-stimulated RAW264.7 cells. Eur. J. Pharmacol..

[35] Murray P.J., Allen J.E., Biswas S.K., Fisher E.A., Gilroy D.W., Goerdt S., Gordon S., Hamilton J.A., Ivashkiv L.B., Lawrence T. (2014). Macrophage activation and polarization: Nomenclature and experimental guidelines. Immunity.

[36] Suleria H.A.R., Addepalli R., Masci P., Gobe G., Osborne S.A. (2017). In vitro anti-inflammatory activities of blacklip abalone (*Haliotis rubra*) in RAW 264.7 macrophages. Food Agric. Immunol..

[37] Ahmad T.B., Rudd D., Kotiw M., Liu L., Benkendorff K. (2019). Correlation between fatty acid profile and anti-inflammatory activity in common Australian seafood by-products. Mar. Drugs.

[38] Saito H., Aono H. (2014). Characteristics of lipid and fatty acid of marine gastropod *Turbo cornutus*: High levels of arachidonic and n-3 docosapentaenoic acid. Food Chem..

[39] Maciejewska-Markiewicz D., Stachowska E., Hawrylkowicz V., Stachowska L., Prowans P. (2021). The role of resolvins, protectins, marensins in non-alcoholic fatty liver disease (NAFLD). Biomolecules.

[40] Manickam M., Meenakshisundaram S., Pillaiyar T. (2021). Activating endogenous resolution pathways by soluble epoxide hydrolase inhibitors for the management of COVID-19. Arch. Pharm..

