# Supplementary material for: Anti-Inflammatory Effect of Turbo cornutus Viscera Ethanolic Extract against Lipopolysaccharide-Stimulated Inflammatory Response via the Regulation of the JNK/NF-kB Signaling Pathway in Murine Macrophage RAW 264.7 Cells and a Zebrafish Model: A Preliminary Study
Source: Foods. 2022 Jan 27;11(3):364. doi: 10.3390/foods11030364 (PMC8834147; doi:10.3390/foods11030364)
Supplement: Supplementary file 1 [file foods-11-00364-s001.zip › foods-1459009-supplementary.pdf]

Supplementary materials

Table S1. Detection of bacteria counts and Escherichia coli in *Turbo cornutus* viscera ethanol extract (TVE)

	CFU/mL
Bacterial count	7
Escherichia coli	ND

ND: not detected

(a) (b)

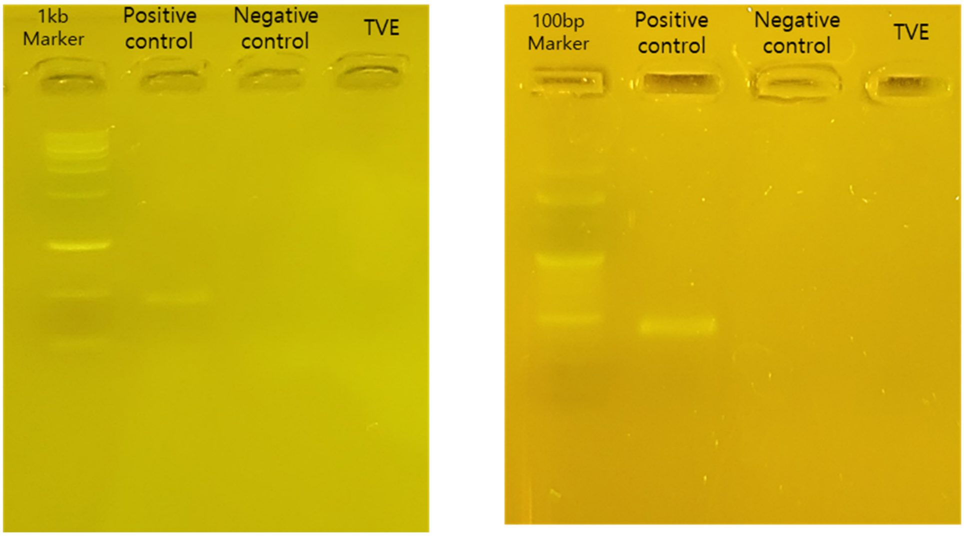

Figure S1. Detection of (a) bacteria and (b) fungal in *Turbo cornutus* viscera ethanol extract (TVE)
